# Right Ventricular Free Wall Rupture Due to Displaced Automatic Implantable Cardioverter Defibrillator (AICD) Lead

**DOI:** 10.7759/cureus.53146

**Published:** 2024-01-29

**Authors:** Abhinav K Rao, Craig Herrforth, Angeli Patel, Kunaal Patel, Brittany Lyons

**Affiliations:** 1 Internal Medicine, Trident Medical Center, North Charleston, USA

**Keywords:** cardiac device implantation, displaced lead, right ventricle, free wall rupture, aicd

## Abstract

The implantation of an implantable cardioverter defibrillator (ICD) carries a risk for major complications, one of which is ventricular free wall rupture secondary to a lead perforation. This known complication, although rare, has estimated incidence rates between 0.1% and 3%. Predictive factors of such an event include temporary leads, steroid use, active fixation leads, low body mass index (<20 kg/m^2^), age greater than 80 years, female gender, and concurrent anticoagulation. Right ventricular systolic pressure >35 mmHg is considered a protective factor likely due to associated right ventricular hypertrophy. We present a case of a 73-year-old female with a history of aortic stenosis status post-transcatheter aortic valve replacement (TAVR) and atrial fibrillation (AFib) who met the criteria for an ICD after suffering ventricular fibrillation arrest (after TAVR procedure) ultimately resulting in lead perforation.

## Introduction

A rare complication that can occur after cardiac device implantation is cardiac perforation, with estimated incidence rates of 1-3%. Perforation is more likely to occur after right ventricle lead placement, due to the low-pressure system, thin wall, and anterior location of the right ventricle [1,2]. Here, we report a case of acute right ventricular free wall rupture due to ventricular intracardiac (ICD) lead perforation in a 73-year-old female that occurred within one day after implantation. Prompt diagnosis and treatment allowed for appropriate operative intervention and patient improvement.

## Case presentation

The patient is a 73-year-old female with a history of paroxysmal atrial fibrillation (PAF), chronic obstructive lung disease (COPD) on 3 L oxygen at home, heart failure with preserved ejection fraction (HFpEF), aortic stenosis treated recently with transcatheter aortic valve replacement (TAVR) with subsequent ventricular fibrillation arrest of unknown etiology eight days later. She underwent emergent left heart catheterization that revealed normal coronary arteries and placement of a dual-chamber automatic implantable cardioverter defibrillator (AICD) at another facility.

She presented to the emergency room within one day of discharge from the facility that placed the AICD with complaints of generalized weakness, shortness of breath, and productive cough. The patient was previously on anticoagulation with apixaban 5 mg twice daily. However, the patient mentioned discontinuing this medication since discharge for unclear reasons. Her other home cardiac medications included lopressor 25 mg twice daily, aspirin 81 mg daily, and rosuvastatin 40 daily. Her COPD medications included ipratropium/albuterol 2-100 mcg/act 1 puff q6h PRN, tiotropium bromide 2.5 mcg/act 2 puffs inhaled QAM, and fluticasone Diskus 44 mcg/act 2 puffs inhaled BID. She denied any recent steroid use for COPD exacerbations. In the emergency department, her blood pressure was 120/75 mmHg, heart rate was 127 beats per minute, respiratory rate was 20 breaths per minute, and she was saturating at 99% on 4 L oxygen. Her labs were notable for sodium of 133 mEq/L, Cl of 93 mEq/L, BUN of 22 mg/dL, glucose of 121 mg/dL, flat troponins 0.06 ng/mL then 0.07 ng/mL, normal WBC of 10.5 k/μL with 80.1% neutrophils, and hemoglobin of 9.8 g/dL (Table 1).

**Table 1:** Laboratory data on admission. Labs on admission were notable for a hemoglobin of 9.8 g/dL, in addition to mild hyponatremia, hypochloremia, hyperglycemia, normal lactic acid, normal WBC count with left shift, and serially flat troponins. BUN: blood urea nitrogen; AST: aspartate transaminase; ALT: alanine transaminase; TSH: thyroid-stimulating hormone; HCT: hematocrit

Chemistry	Values	Reference range
Sodium	133 mEq/L	136-145 mEq/L
Potassium	3.8 mEq/L	3.6-5.1 mEq/L
Chloride	93 mEq/L	101-111 mEq/L
Carbon dioxide	30 mEq/L	22-32 mEq/L
Anion gap	10	3-13
BUN	22 mg/dL	6-20 mg/dL
Creatinine	0.8 mg/dL	0.4-1.0 mg/dL
Glucose	121 mg/dL	70-100 mg/dL
Lactic acid	0.7 mEq/L	0.5-2.0 mEq/L
Calcium	9.2 mg/dL	8.9-10.3 mg/dL
Total bilirubin	0.9 mg/dL	<1.0 mg/dL
AST	30 U/L	35 U/L
ALT	35 U/L	10-63 U/L
Alkaline phosphatase	72 U/L	32-101 U/L
Troponin	0.06 ng/mL	<0.04 ng/mL
Albumin	3.7 g/dL	3.4-4.8 g/dL
TSH	1.98 mIU/mL	0.34-5.60 mIU/mL
Free T4	1.2 ng/dL	0.6-1.1 ng/dL
Hematology		
WBC	10.5 k/μL	4.0-10.9 k/μL
Hb	9.8 g/dL	11.5-14.5 g/dL
HCT	29.8%	34.0-43.0%
Platelets	258 k/mm^3^	135-350 k/mm^3^

Computed tomography (CT) with and without contrast showed no evidence of pulmonary thromboembolism but did show moderate alveolar opacities and reticular nodular opacities in the upper lobes, right greater than left, and suspicious for underlying pneumonia. A moderate pericardial effusion was also noted. An electrocardiogram (EKG) revealed atrial fibrillation with a rapid ventricular response. Her home metoprolol tartrate 25 twice daily for her chronic PAF was resumed with improvement in heart rate to 100 beats per minute. She was started on antibiotic coverage for hospital-acquired pneumonia with vancomycin 15 mg/kg IV q12h and cefepime 1 g q12h given her recent hospitalization.

**Figure 1:**
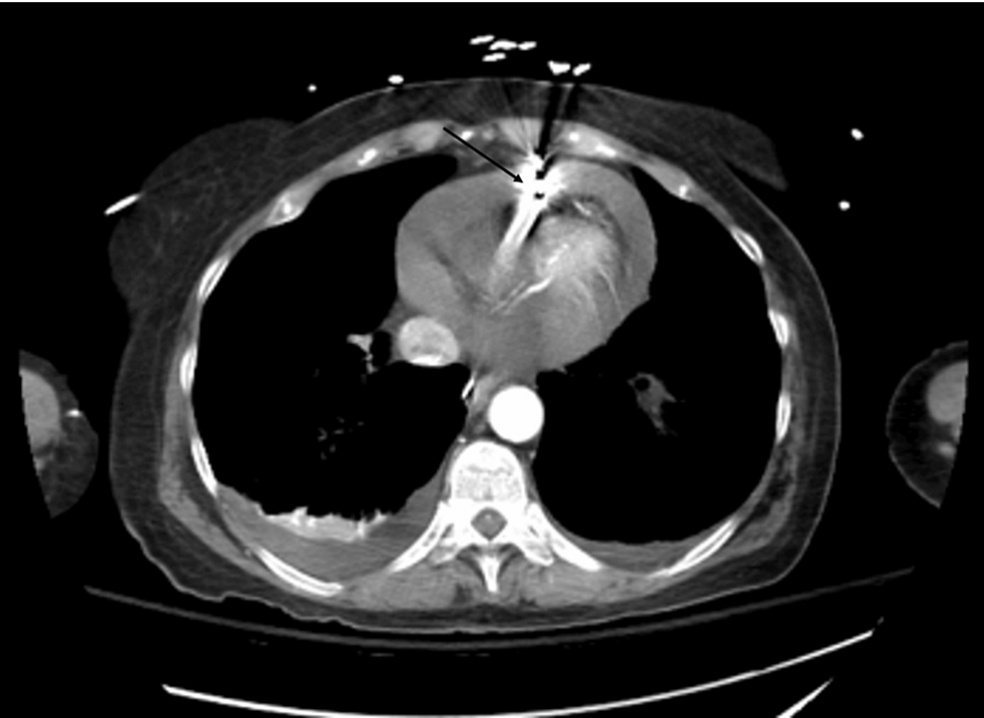
Pacemaker wire traversing pericardium with moderate pericardial effusion. Bilateral pleural effusions are also noted.

On day four of hospitalization, the patient developed symptoms of periarrest with systolic blood pressure in the 60s mmHg. The patient was resuscitated by the critical care team and intubated. A central line was placed, and the patient was started on Levophed and vasopressin for the treatment of cardiogenic shock. She was also intubated for airway protection. An echocardiogram revealed an ejection fraction (EF) of 65-70% and significant mitral valve inflow variation, which was suggestive of possible cardiac tamponade of hemodynamic significance. Her right ventricle (RV) lead was noted to be in an unusual position raising concerns for perforations. CT scan showed bilateral pleural effusions with moderate pericardial effusion that had progressed from the prior study. The pacemaker wire was noted to be traversing the pericardium (Figure 1). Hemoglobin was noted to be 8.9 g/dL with a prothrombin time (PT) of 18.7 seconds and an international normalized ratio (INR) of 1.59. A right heart catheterization was completed which showed a low cardiac index of 1.67 and normal filling pressures. Her right ventricular pressure was noted to be 20/8 mmHg.

Cardiothoracic surgery was consulted, and the plan was made for pericardial window and evacuation. On hospital day six, she was taken to the operative room (OR), and tamponade was treated with subxiphoid pericardial window, evacuation of a large clot from the pericardium tamponade, and right ventricular repair. Specifically, the RV lead was cut to allow it to retract into the RV, and the area of the RV that was continuing to bleed was repaired. Her pressors were switched to phenylephrine on hospital day seven due to her course being complicated by atrial fibrillation with rapid ventricular rate (RVR). She was also started on milrinone on hospital day nine. Her phenylephrine was weaned off on hospital day 10 and milrinone was weaned off on hospital day 11. Her antibiotics were transitioned to ampicillin/sulbactam 1.5 g q6h on hospital day two, and she was treated for seven days. She was also successfully extubated on hospital day eight.

Right ventricular lead revision with removal and replacement was eventually completed once the patient clinically improved. She was eventually transferred to the hospitalist service and discharged after a 23-day hospital stay with significant improvement. Her home metoprolol tartrate 25 mg twice daily was discontinued upon discharge; however, the rest of her medications remained unchanged. Her chest x-ray (CXR) six days prior to discharge revealed a normal cardiac silhouette. At a two-month follow-up, the patient was doing well and appeared at her baseline state of health. Her serial CXR at this time revealed no further evidence of pericardial fluid.

## Discussion

Implantable cardioverter defibrillator (ICD) implantation is associated with the risk of major complications, one of which is ventricular free wall rupture secondary to lead perforation [1]. Several predictive factors for lead perforation have been identified, including the use of temporary leads, steroid use, active fixation leads, low body mass index (<20 kg/m^2^), age greater than 80 years, female gender, and concurrent anticoagulation [1,2]. It is noteworthy that a right ventricular systolic pressure greater than 35 mmHg is considered a protective factor, possibly due to the associated right ventricular hypertrophy [3]. Despite the occurrence of lead perforation, most patients remain asymptomatic [4].

Cardiac perforation is a rare, potentially life-threatening, complication of pacemaker implantation with an estimated incidence rate between 0.1% and 3% [5-8]. The complications related to cardiac perforation generally decrease over time and are categorized based on the time frame between implantation and detection. Acute perforation occurs within 24 hours, subacute occurs within one month, and perforation after one month is considered chronic [7,8]. Affected sites include the walls of the large veins, atria, or ventricles, with the thinner RV apex being more common [8].

The mechanism of wall perforation is related to the physical properties of the lead and over-torquing of the leads during implantation. The lead dimensions are very thin with a heavy tip, which is thought to increase wall stress [9]. Regarding technique, the operator’s torque was likely all in the tip of the lead with a non-retractable screw, further propagating stress to the deeper layers of the myocardium [10]. Resisting from screwing a lead too deeply and reducing slack are modifiable technical components that may prevent this complication from occurring [10].

Chest x-ray and echocardiography (ECHO) are inexpensive and convenient, though may not always reveal the diagnosis. If the lead extends beyond the cardiac silhouette, a chest x-ray can be diagnostic. A lateral view more accurately localizes the pacemaker lead position. The added benefit of the chest x-ray is to detect extracardiac complications including pleural or pericardial effusion and pneumothorax [11]. ECHO can also help detect a pacemaker lead in the pericardium and pericardial effusion; however, like the chest x-ray, it may not correctly locate the pacemaker lead tip [11,12]. Thus, if suspecting a pacemaker lead perforation that is not clearly evident on chest x-ray or ECHO, a CT scan should be ordered. This is the most accurate modality in assessing pacemaker lead placement with the added benefit of confirming extracardiac disease [11]. However, it should be noted that image artifacts may rarely lead to misinterpretation of the position of pacemaker wires [10].

Pacing irregularities can be indicative, as demonstrated in a scenario involving left ventricular free wall perforation caused by a right ventricular pacemaker. Initially, there might be pacing failure due to the lead penetrating through the septum [10]. Subsequently, intermittent pacing in the left ventricular wall might occur after septal penetration, followed by eventual penetration through the left ventricular free wall, marked by complete pacing failure. However, it's essential to note that the absence of sensing, pacing failure, and even normal function during device interrogation do not conclusively rule out perforation [10].

The primary treatment involves evacuating any pericardial fluid if it is present and repairing the ventricular damage through cardiothoracic surgery. Once the patient is stable, revising the ventricular lead becomes crucial for comprehensive management [4].

## Conclusions

This study presents a rare case of a 73-year-old female with a history of aortic stenosis status post-TAVR and atrial fibrillation who presented with right ventricular free wall rupture following a displaced AICD lead. Given its rarity, her symptoms were not discovered until day eight when she went into cardiogenic shock. After diagnosis, prompt treatment with pericardial fluid evacuation, ventricular repair, and lead revision led to patient improvement. Considering the associated morbidity and mortality linked with AICD displacement, clinicians should maintain awareness of this possibility in patients who have recently undergone AICD placement.
